# Parental Phubbing Behavior and Adolescents’ Online Gaming Time: The Mediating Role of Electronic Health Literacy

**DOI:** 10.3390/bs14100925

**Published:** 2024-10-10

**Authors:** Jinnan Liu, Tao Xie, Ying Mao

**Affiliations:** School of Public Policy and Administration, Xi’an Jiaotong University, Xi’an 710049, China; kennanliu@stu.xjtu.edu.cn

**Keywords:** parental phubbing behavior, online gaming time, electronic health literacy

## Abstract

Objective: The purpose of this study is to explore the relationship between parents’ phubbing behavior and children’s online gaming time. More importantly, we investigated the mediating role of electronic health literacy in this association. Methods: A total of 1200 questionnaires were sent out and 1130 effective questionnaires were returned, an effective recovery of 94.17%. As some students did not answer part of the EHL survey, eventually only 867 students were included in this study based on the selected independent variables and the degree of questionnaire completion. Both tools used for measuring parental phubbing and electronic health literacy passed confirmatory factor analyses. To analyze the mediating effect of electronic health literacy on parental phubbing behavior and online gaming time, 5000 datapoints were sampled using the Bootstrapping method. Results: The results indicated that parents’ phubbing was positively related to their children’s online gaming time, which was mediated by electronic health literacy, parental phubbing is positively and significantly correlated with electronic health literacy, and electronic health literacy is negatively and significantly correlated with online gaming time among adolescents. Conclusions: Electronic health literacy partially mediates the relationship between parental phubbing behavior and adolescent online gaming time, and Chinese students’ electronic health literacy is a two-dimensional structure consisting of health information acquisition and application. This study is the first empirical study to explore the relationship between parental phubbing behavior and the amount of time adolescents spend playing games online.

## 1. Introduction

The number of underage Internet users in China has been increasing, with the number having exceeded 193 million in 2022. From 2018 to 2022, the Internet penetration rate of minors grew from 93.7% to 97.2%, reaching a saturation state. Trends have clearly shown that minors are using the Internet at younger ages, with the Internet penetration rate for minors at primary school ages having increased from 89.5% to 95.1% over the past five years. Playing games on the Internet is the main choice of online activity for leisure and entertainment in minors: the proportion of underaged Internet users playing computer games is 24.0%, playing mobile phone games is 62.8%, and the proportion of those who regularly engage in these activities is 67.8%. Worth noting is that, with the increasing popularity of online games and short videos among the underaged group, parents have become more worried about the possible adverse effects of these online entertainment applications on their children. A survey of parents and teachers found that 51.8% of parents and 69.9% of teachers (mainly classroom teachers) believed that Internet addiction caused by online games and short videos was the most important problem of Internet use by minors that needs to be addressed [1]. The underaged group has become the main target audience of Internet games [1], and Internet addiction is common among adolescents [2].

Addictions to online games in adolescents can cause not only sleep disorders [3,4], inattention [5], interpersonal relationship damage [3], and other consequences but also anxiety [3], depression [4,6], aggressive and violent behavior [7], impaired social function [5,8], and even suicidal ideation and behavior [9], which can seriously affect their familial, social, educational, and other relationships [3,5,10]. Primary and secondary school students addicted to online games mainly spend a lot of time on mobile games [11,12]. At the same time, over the course of repeated game behavior, primary and secondary school students addicted to mobile games need to continue playing online games for prolonged periods of time to obtain the same levels of psychological satisfaction.

Therefore, in order to protect the healthy growth of minors and to reduce the negative impact of online games on students, China’s national authorities have intensified efforts in its governance. Since August 2021, governing departments such as the National Press and Publication Administration and the Ministry of Education of the People’s Republic of China have issued notices such as the “Notice on further strict management to prevent minors from indulging in online games” and “To further strengthen the prevention of primary and secondary students addicted to online games management notice” to further strengthen policies for preventing Internet addictions in primary and middle school students, such as those addressing the strict control of minors’ online game time, the prevention of addictions to online games in minors, and the promotion of the healthy growth of minors. Hence, strict control of minors’ online game time is an important means of preventing addiction to online games.

Phubbing is a new term synthesized from “phone” and “snubbing”. It refers to the behavior of an individual who is in a social situation but too busy using or playing with their mobile phone to care about others, also known as snubbing, or their surroundings [13,14]. Parental phubbing is specifically the negative parent–child interaction that results when parents use or are distracted by their smartphones when interacting with their children to a degree that is neglectful [15]. In other words, it refers to parents being too busy with their mobile phones to pay attention to their children. The neglect of children may cause a series of negative consequences, such as the child’s independence, the development of depression [15,16,17], academic burnout [18], a dependence on mobile phones [16,17], and damage to the child’s interpersonal skills [16]. Therefore, as phubbing has become a common phenomenon, studies on its effects have garnered more and more attention [19,20,21].

Current research has yet to fully establish the direct and indirect relationships between parents’ phubbing behavior and children’s online playtime. Therefore, this study addresses the critical gap in the literature by linking parents’ phubbing behavior with adolescents’ online activities, a connection that is underexplored but increasingly relevant in the digital age. This study also contributes to the broader discourse on digital parenting and its repercussions on youth behavior and well-being. Furthermore, by focusing on electronic health literacy, this research highlights the potential of health informatics in influencing youth behavior positively, which is particularly pertinent as electronic health literacy becomes an essential skill in navigating not only health information but also other aspects of digital life.

The primary goal of our investigation is to explore the impact of parental phubbing—parents’ engagement with their smartphones to the point of exclusion of face-to-face interactions with their children—on the amount of time adolescents spend engaging in online gaming. Additionally, this study examines the mediating role of electronic health literacy in moderating this relationship.

## 2. Literature Review and Research Hypothesis

### 2.1. Parental Phubbing and Adolescents’ Online Gaming Time

Several studies have suggested that parents’ phubbing behavior may indirectly increase adolescents’ online gaming time, although the findings need to be further validated by more direct evidence. For example, Socialization Theory points out that not only values, sociality, emotions, and other psychological traits [22,23,24,25] but also behavioral patterns [26,27] and parents’ phubbing behavior [24] are transmitted between generations. In other words, the amount of time that parents spend online can be passed on to their children.

Technological Determinism argues that technological development drives social structure and cultural values, suggesting that increasing digital interactions is an inevitable outcome of technological advancement [28]. Contrarily, Social Shaping of Technology advocates that technology is largely shaped by social forces and human choices [29]. In the context of our study, this theory emphasizes the role of familial and societal norms over the inherent qualities of a technology itself. Similarly, Social Control Theory points out that parental monitoring is an important way to achieve social control [30]. Parents regulate adolescents’ behaviors by means of monitoring, such as knowing about, paying attention to, restricting, and guiding them in their participation in activities. For example, good parental monitoring can reduce Internet addiction [31]. Under parental supervision, parents restrict the activities their children engage in, directly limiting their use of mobile phones and computers and thus reducing online gaming time. However, phubber parents tend to relax the monitoring of their children, resulting in neglect of the children. Children who cannot obtain their parents’ attention may turn to online games for comfort and thus increase their playtime. The Theory of Parental Mediation suggests that parental guidance and mediation play critical roles in shaping adolescents’ digital habits [32]. In the current study, we explore the relationships between parental phubbing and adolescents’ online gaming time and, based on the results of previous studies, propose the following hypotheses:

**H1.**  *Parental phubbing is positively associated with adolescent’ online gaming time.*

Parents’ behavioral patterns and communication styles have a significant impact on their children’s health behavior [33]. McDaniel and Radesky’s study found that “technological distractions” in the home may have a detrimental effect on children’s cognitive and emotional development [34]. Such distractions reduce face-to-face communication between family members, which may affect children’s ability to assess and process health information, a key component of eHealth literacy. The family environment is a key factor in the development of eHealth literacy in children. Children are more likely to develop the ability to effectively use eHealth resources in an environment that supports and promotes healthy discussions [35]. However, parental phubbing behaviors may create an environment that lacks this support, thereby inhibiting the development of eHealth literacy. Parental behavioral patterns directly influence their children’s information-seeking behavior. Research has shown that children have higher levels of information literacy when parents actively guide them on how to find and evaluate information online [36]. Thus, parental cold-shouldering behaviors may indirectly lead to lower eHealth literacy in children by reducing such guidance. Based on the above evidence, we can infer that parental phubbing may have a negative impact on children’s eHealth literacy.

**H2.**  *Parental phubbing is negatively related to electronic health literacy.*

### 2.2. Mediating Role of Electronic Health Literacy

Although parental phubbing may significantly increase the amount of time adolescents spend playing online games, note that not all adolescents prolong their online gaming time when faced with parental phubbing. As electronic health literacy is considered an important factor in predicting the behavior of students seeking electronic health information [35], we suggest that electronic health literacy plays a mediating role in the relationship between parental phubbing behavior and adolescents’ online gaming time. Health literacy refers to the ability of individuals to obtain and understand health information and make correct health decisions using this health information [37]. Obviously, electronic health literacy is a concept consisting of both health literacy and electronic health information [38]: it is the ability of individuals to obtain, understand, judge, and use health information from electronic resources to solve their own health problems [36]. However, to the best of our knowledge, no studies have shown whether electronic health literacy mediates the relationship between parental phubbing and teen online gaming time.

The concept of electronic health literacy has been gradually expanded in recent years, and, in particular, the understanding of its structure has evolved from a single dimension to a multidimensional one. Recent studies have proposed a two-dimensional structural model of eHealth literacy, a model that not only includes the traditional skills and knowledge dimensions but also emphasizes the importance of situational awareness [35]. The skills and knowledge dimension focuses on an individual’s ability to access, understand, and evaluate health information, which is the foundation of eHealth literacy [37]. Some studies found that the eHEALS with a two-factor structure, consisting of information acquisition and information application, is more convincing than a single-factor structure [38,39].

It can be seen from the above that electronic health literacy is the ability to obtain and use health knowledge through electronic sources to maintain and improve one’s health, which is essentially the acquisition and application of health information. One manifestation of parents’ phubbing behavior is their excessive use of smart phones. Therefore, according to the Theory of Intergenerational Transmission [22,23], we can infer that parents’ use of smart phones will be passed on to their children, and these children may develop a phubbing tendency as well by imitating their parents’ behavior [20] and thus increase their use of smart phones. Adolescents can gain access to a huge amount of information, including health information, through smartphones. For example, studies have found that both boys and girls aged 16–17 consider the Internet to be their main source of information [40]. Similarly, parents’ phubbing behavior can be considered an unhealthy behavior, and adolescents generally regard parents as one of their main sources of health information [41], so unhealthy parental technology use may also affect children’s relationship with health information [42]. Previous studies have also demonstrated a positive correlation between excessive Internet use and mobile phone use by parents and children [26,43,44].

Through the above systematic review of health literacy, we found that the time spent playing online games is one of the important factors affecting the health literacy of adolescents [45,46]. Students spend a lot of time and energy on playing games, which occupies their study time and hinders improvements in their health literacy. Therefore, students who play games for a long time, which indicates relatively weaker self-control, often cannot effectively obtain and utilize their health knowledge and skills [47,48]. Other studies have demonstrated a negative correlation between health literacy and the amount of time students spend online [49]. Studies have also shown that students with low health literacy are more likely to be addicted to the Internet than students with high health literacy [50]. Therefore, we believe that electronic health literacy is negatively correlated with the online gaming time of adolescents.

**H3.**  *Electronic health literacy is negatively related to adolescents’ online gaming time.*

**H4.**  *Electronic health literacy mediates the relationships between parental phubbing and adolescents’ online gaming time.*

**Figure 1:**
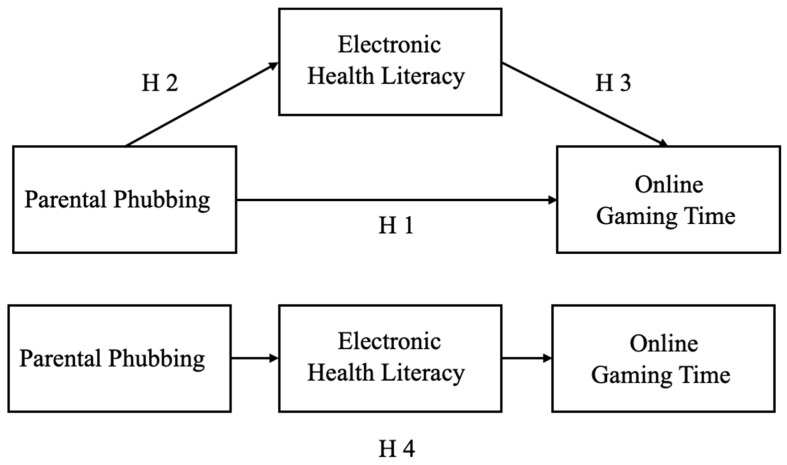
The mediation model.

As seen in the above, no research has proven the direct and indirect relationships between parents’ phubbing behavior and children’s online gaming time. Hence, the purpose of this study is to explore the relationship between parents’ phubbing behavior and children’s online gaming time. More importantly, we investigate the mediating role of electronic health literacy in this association. Accordingly, we propose the four hypotheses presented in Figure 1:

## 3. Materials and Methods

### 3.1. Study Design and Procedures

This study adopts the stratified cluster random sampling method to select primary and secondary school students in Shaanxi Province, China, as the research objects. Two primary schools, two junior high schools, and two senior high schools were randomly selected, and four classes were randomly selected from each school. A total of 1200 questionnaires were sent out, and 1130 effective questionnaires were returned, an effective recovery of 94.17%. As some students did not answer part of the EHL survey, eventually, only 867 students were included in this study based on the selected independent variables and the degree of questionnaire completion.

This study was approved by the Biomedical Ethics Committee, School of Medicine, Xi’an Jiaotong University (No. 2021–1525). After providing details about our study, all participants in this study, or their legal guardians for participants below 16 years of age, gave their informed consent and voluntarily participated in the survey. The data were collected from two primary schools, two junior high schools, and two senior high schools in Shaanxi Province, China, between June and August 2021.

### 3.2. Measures

#### 3.2.1. Parental Phubbing

The Parental Phubbing Behavior Scale (PPB) is a scale based on the Partner Phubbing Scale revised by Ding et al. [51]. The Partner Phubbing Scale (PPS), proposed by James and Meredith [19], mainly discusses the behavior of partner phubbing, which refers to individuals often ignoring or neglecting others to use or play with their mobile phones while engaging with others or in social situations. The PPB is mainly used for adolescents, with nine items in total: PPB1. When I eat with my parents, they use their mobile phones; PPB2. When I spend time with my parents, they keep their mobile phones out of sight; PPB3. When I spend time with my parents, they keep their mobile phones in plain sight; PPB4. When the phone rings, they check it immediately, even when we are in the middle of a conversation; PPB5. When I am talking to my parents, they will stare at their mobile phones without taking their eyes off it; PPB6. I will use my mobile phone with my parents in my spare time; PPB7. When I am talking with my parents, they do not use their mobile phones (reverse question); PPB8. When I go out with my parents, they use their mobile phones; and PPB9. My parents check their mobile phones from time to time while I am chatting with them. Subjects’ feelings, reactions, and degree of identification with the statements were taken as evaluation indexes, and the five-point scale grading method was adopted. The standard responses were 1—never; 2—little; 3—sometimes; 4—often; and 5—always. In the current study, Cronbach’s alpha for the PPB was 0.835.

#### 3.2.2. Electronic Health Literacy

The eHealth Literacy Scale (eHEALS), prepared by Cameron and Harvey [52], is the first and currently most commonly used EHL assessment tool, mainly measuring the self-perception skills of netizens when they seek and apply online health knowledge. The eHEALS consists of eight items rated on a five-point scale (1 = strongly disagree; 5 = strongly agree): EHL1. I know where to find helpful health resources on the Internet; EHL 2. I know how to find helpful health resources on the Internet; EHL 3. I know what health resources are available on the Internet; EHL 4. I know how to use the health information I find on the Internet to help me; EHL 5. I know how to use the Internet to answer my questions about health; EHL 6. I feel confident in using information from the Internet to make health decisions; EHL 7. I have the skills I need to evaluate the health resources I find on the Internet; and EHL 8. I can tell high-quality health resources from low-quality health resources on the Internet. In the current study, Cronbach’s alpha for the eHEALS was 0.909.

#### 3.2.3. Online Gaming Time

The variable online gaming time was measured using the responses to one question: “How many hours per week do you play online games?” OGT was treated as a continuous variable.

#### 3.2.4. Demographic Characteristics

The questionnaire gathered information about gender, age, race, stage of education, whether they were an only child, and place of birth.

### 3.3. Statistical Analysis

All statistical analyses were performed using SPSS Statistics version 26.0 (IBM Corporation, Armonk, NY, USA) and AMOS version 23.0 for Windows (IBM Corporation, Armonk, NY, USA). The statistical methodology of the research consisted of the following stages: (i) We employed a confirmatory factor analysis (CFA) to validate the measurement models for our key constructs. This method helped us ensure that our items reliably reflected the constructs they were intended to measure. (ii) Structural equation modeling (SEM) was used to test the hypothesized relationships between the constructs. This approach not only allowed us to examine the direct paths, such as the impact of parental phubbing on adolescents’ online gaming time, but also enabled us to analyze the mediating role of EHL in this dynamic. (iii) To analyze the mediating effect of electronic health literacy on parental phubbing behavior and online gaming time, 5000 datapoints were sampled using the Bootstrapping method. To ensure the reliability of our findings, a statistical power analysis was conducted to assess the adequacy of the sample size [53]. To calculate the statistical power, we used Python’s statsmodels, with the two-tailed significance level set at 0.05 [54].

## 4. Results

### 4.1. Sample Characteristics

Of the participants, 421 were male (48.56%), 446 were female (51.44%), their mean age was 13.71 (SD = 2.93), 277 were in primary school (31.95%), 267 were in junior high school (30.80%), 323 were in high school (37.25%), and 99.54% were ethnically Han. Most of the participants lived in rural areas (60.44%). The mean score for online gaming time was 1.97 (SD = 2.13), with a range from 0 to 9 (see Table 1). The analysis revealed that the statistical power was 1.0, indicating that the sample size was sufficient to detect the observed effect.

### 4.2. Confirmatory Factor Analysis

Before testing the association, a CFA was used to test the data fit for the measurements. The CFA results showed that the eHEALS included two latent variables (health information acquisition and application) and six observed variables, while the PPB had one latent variable and six observed variables. The fit indices of the CFA were as follows: *X*^2^ = 242.676; df = 51; *X*^2^/df = 4.758; GFI = 0.955; AGFI = 0.931; CFI = 0.959; TLI = 0.947; and RMSEA = 0.066. The composite reliability of information acquisition, information application, and the PPB was 0.843, 0.861, and 0.850, respectively, and Cronbach’s alpha was 0.841, 0.857, and 0.846, respectively. Factor loading was shown in Table 2. The results had good reliability and validity in the current study.

### 4.3. Parameter Estimates

As shown in the path coefficient in Table 3, we can see that parental phubbing behavior is positively and significantly correlated with adolescents’ information acquisition and positively and significantly correlated with adolescents’ information application. Parental phubbing behavior is positively correlated with the amount of time adolescents spend playing online games every week. Adolescents’ information acquisition and information application are negatively correlated with their weekly playtime for online games.

### 4.4. Testing for the Mediation Effect of Electronic Health Literacy

**Figure 2:**
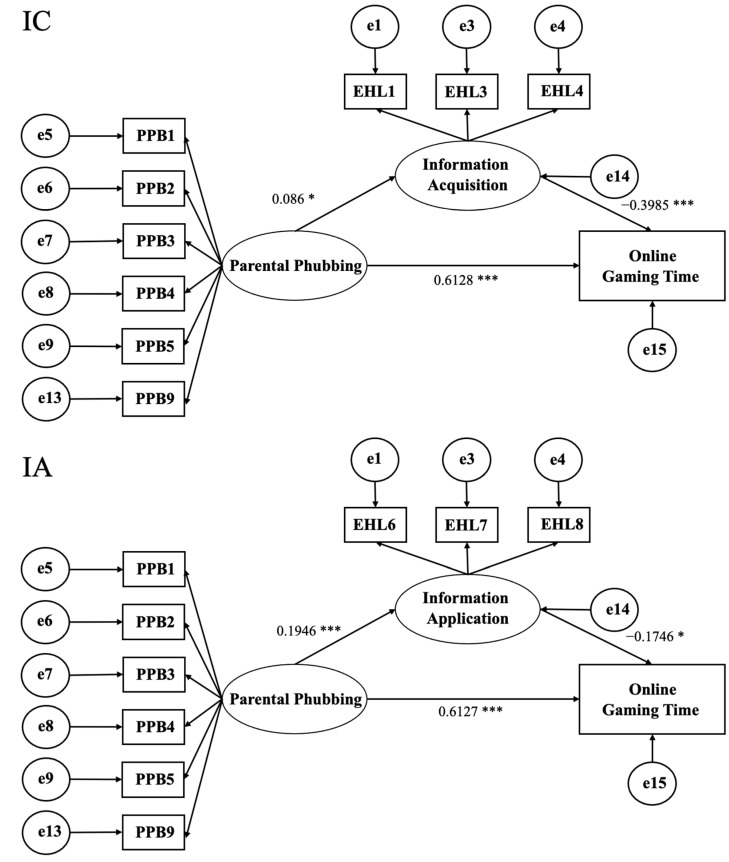
The mediation effect of electronic health literacy. * significant at the 0.05 level (two-tailed); *** significant at the 0.001 level (two-tailed).

After the confirmatory factor analysis, based on the theoretical framework, structural equation models were constructed with information acquisition and application as separate mediators (see Figure 2), with good degrees of fit (see Table 4). The mediating effects of this study were verified using the Bootstrapping methodand via sampling 5000 times, and 95% bootstrap confidence intervals (CIs) were calculated. This method can estimate the standard error and non-standardized coefficient of indirect effects, as well as the confidence interval. A confidence interval that does not contain zero indicated that a mediation effect existed.

As shown in Table 5, the non-standardized coefficient of the indirect effect of the information acquisition-mediated model was −0.0343, and the bias-corrected 95% CI and percentile 95% CI were [−0.0861, 0.0044] and [−0.0834, 0.0074], respectively, which included 0. Thus, the two-tail tests with bias-corrected and percentile 95% CIs were not significant, indicating that information acquisition did not have a mediating effect. The non-standardized coefficient of the indirect effect of the information application-mediated model was −0.034, and the bias-corrected 95% CI and percentile 95% CI were [−0.0792, −0.007] and [−0.0743, −0.0047], respectively, which did not include 0. Thus, the two-tailed tests with the bias-corrected and percentile 95% CIs were significant, indicating that information application had a mediating effect. Additionally, we found that the direct effect was significant in this model, so information application had a partial mediating effect.

## 5. Discussion

### 5.1. Principle Findings

To our knowledge, although an increasing number of studies have focused on the negative effects of parental phubbing behavior, the potential mechanisms mediating the relationship between parental phubbing behavior and adolescents’ online gaming time remain unclear. In this study, our findings indicate that (1) parental phubbing significantly and positively correlates with adolescents’ online game time and electronic health literacy; (2) electronic health literacy is significantly correlated with online gaming time among adolescents; and (3) the use of health information partially mediates the relationship between parental phubbing and adolescent online gaming time.

In the contemporary research milieu, where digital engagement is ubiquitous and its effects on mental health are a growing concern, our study provides empirical evidence that can inform educational strategies and public health interventions. This research is vital for developing targeted programs that enhance digital literacy and promote healthier online habits among adolescents, thereby mitigating the potential adverse effects of digital overuse.

Consistent with hypothesis 1, parental phubbing significantly and positively correlated with adolescents’ online game time; the discovery in our forefathers’ research was basically identical [55]. Research suggests that when adolescents perceive that their parents are frequently distracted by their phones, they may compensate for this lack of attention by spending more time on online activities, such as gaming [56]. For example, a study found that parental phubbing behavior is positively correlated with adolescents’ problematic smartphone use, including excessive time spent on online games [24,56]. In situations where parental engagement is reduced due to phubbing, adolescents may turn to online games as an alternative means of engagement and interaction, exacerbating their screen time and potential for gaming addiction [25,27]. Therefore, we can reasonably assume that parental phubbing will significantly increase the frequency of online gaming among adolescents. In other words, teens exposed to parental phubbing will increase their online gaming time. Parental phubbing often leaves children out of the spotlight due to the focus being on mobile devices. These adolescents often receive little attention from their parents and thus may turn to online games for comfort. At the same time, according to the Theory of Intergenerational Transmission, such phubbing behavior of parents will be passed on to their children, who may then become addicted to mobile online games.

During our research, we found a two-factor structure of electronic health literacy—acquisition and application of health information—which is consistent with the research of Renate et al. [57] and Patricia and Jordan [38]. This is also a new discovery among Chinese students. Contrary to our expectations (hypothesis 2), parental phubbing is positively and significantly correlated with electronic health literacy. Specifically, the acquisition and application of health information are positively and significantly correlated with parental phubbing. Adolescents exposed to higher levels of parental phubbing tend to engage more with digital platforms, which may enhance their ability to navigate online spaces, including health information platforms [56].

According to previous studies, phubbing often also brings negative effects, such as depression in children [15,58], academic burnout [59], and dependence on mobile phones [44,60]. However, in this study, we found the opposite: the positive correlation between parental phubbing and electronic health literacy can be explained by the increased time spent in online environments. Adolescents may compensate for the lack of parental engagement by turning to the Internet for information, including health-related content. As they become more self-reliant in acquiring and applying this information, their electronic health literacy improves [24]. Another possible explanation is that schools with a high level of education will teach adolescents how to obtain health information and to stay healthy. Adolescents with health knowledge and skills will pay attention to their own health behaviors and take the initiative to choose a healthier lifestyle even in the face of their parents’ phubbing. Some adolescents even feedback their health knowledge to their elders through intergenerational communication. On the contrary, adolescents with low levels of education will have more behavioral problems and may increase their time spent playing online games in the face of their parents’ phubbing.

Consistent with hypothesis 3, electronic health literacy is negatively and significantly correlated with online gaming time among adolescents. Specifically, the acquisition and application of health information are significantly negatively correlated with the online gaming time of adolescents. This result is consistent with those of previous studies, which demonstrated a negative correlation between health literacy and students’ online gaming time [49]. Adolescents with higher electronic health literacy tend to spend less time on online games, likely because they are more adept at recognizing the negative health impacts of excessive gaming and prioritizing healthier behaviors [24,25]. For example, research has shown that adolescents who are more proficient in seeking, understanding, and applying health information from electronic sources tend to allocate their screen time to health-related activities rather than entertainment such as gaming [24,25]. This negative correlation can be further explained by the fact that adolescents who actively acquire and apply health information are more conscious of the importance of physical and mental well-being, which might lead them to limit time spent on activities like online gaming. The acquisition and practical application of health information—key components of electronic health literacy—equip them with the knowledge to make informed decisions regarding their screen time and health management. Therefore, promoting eHealth literacy could be an effective approach to reducing excessive online gaming among adolescents and encouraging healthier screen habits [27]. The above research results further demonstrate that improving individual electronic health literacy is of great significance for improving adolescent Internet addiction and other health risk behaviors.

It is worth noting that we analyzed the mediating effect of the two factors of electronic health literacy and innovatively found that the application of health information played a mediating role in the relationship between parental phubbing and adolescents’ online gaming time. As expected, the use of health information partially mediated the relationship between parental phubbing and adolescent online gaming time, consistent with hypothesis 4. This is a new finding, and we have not found any studies to prove this mediating effect, which explains why parental phubbing significantly increases online gaming time among adolescents who have low levels of electronic health literacy. In other words, teens with high levels of phubbing from their parents but high levels of health information use are more likely to understand that prolonged online gaming is a behavioral problem and to reduce the duration of such behavior.

### 5.2. Limitations and Future Directions

Some limitations should be noted in this study. First, since this study was designed based on correlation, no causal relationship could be inferred. For example, future studies could be conducted by tracking adolescents’ online time and parents’ phubbing behavior, setting up control and experimental groups, and using causal inference methods (Differences-in-Differences and Regression Discontinuity Design). Second, the measures of parental phubbing behavior in the study were completed by the child/children and online gaming time was used as a single indicating factor. In future studies, we can take the family as a unit, obtain nested data through self-reports by family members (parents and guardians), adopt the multilevel regression method to explore the presence of any hierarchical differences, and add more relevant indicators to build a composite indicator for online game time. Third, this study only included one mediating factor and did not analyze the moderating effect. In the future, health literacy can be used as a moderator to analyze whether a difference exists between adolescents with and without health literacy. Fourth, although the sample in this study included both Chinese primary and secondary school students, no cross-cultural analysis was performed. Future studies can try to explore these relationships in different cultural contexts based on a multigroup analysis. Fifth, this study does not consider what the parents use their smartphones for and how they use their smartphone but only focuses on time spent on their smartphones. To understand the impact of smartphone use, future research should consider the specific activities that parents engage in on their smartphones as a whole (e.g., work, social media browsing, news reading, etc.) and how the nature of these activities affects their children.

## 6. Implications

### 6.1. Theoretical Implications

Our study enriches the existing literature on parent–child relationships via demonstrating a clear linkage between these relationships and the health behavior outcomes in children and adolescents. Specifically, the two-dimensional structure of the eHEALS identified in our research offers a nuanced understanding of how children and adolescents access and evaluate health information online. This contributes to theoretical frameworks via providing empirical support for the significance of digital health literacy in shaping young individuals’ health behaviors, suggesting that future research could further explore the mechanisms through which parent–child interactions influence eHealth literacy.

### 6.2. Practical Implications

From a practical standpoint, the insights gained from this study are pivotal for developing targeted health education programs. Policymakers and educators can use the validated structure of eHEALS as a reliable tool for assessing and enhancing EHL among primary and secondary school students. By incorporating these findings, educational interventions can be tailored to improve students’ capacities to critically assess health information. This is particularly crucial in today’s digital age, where children and adolescents are increasingly reliant on online sources for health-related knowledge. Furthermore, understanding the role of parent–child dynamics in this context can guide parental involvement in health education, thereby fostering healthier decision-making behaviors among youths.

Overall, these implications highlight the need for continued investigation into the factors influencing EHL and underscore the potential of informed educational practices to positively impact adolescent health behaviors.

## 7. Conclusions

In summary, this study provides some contributions despite the limitations mentioned above; this study is the first empirical study to explore the relationship between parental phubbing behavior and the amount of time adolescents spend playing games online. These findings suggest that electronic health literacy partially mediates the relationship between parental phubbing behavior and adolescent online gaming time. Another contribution of this study is the discovery that Chinese students’ electronic health literacy is a two-dimensional structure consisting of health information acquisition and application.

## Figures and Tables

**Table 1:** Sample characteristics.

Variable	Levels	N	%
Gender	Male	421	48.56
	Female	446	51.44
Race	Han	862	99.42
	Non-Han	4	0.46
Stage of education	Primary school	277	31.95
	Junior high school	267	30.8
	High school	323	37.25
Only child	Yes	70	8.08
	No	797	91.92
Place of birth	City	343	39.56
	Rural	524	60.44

**Table 2:** Factor loading.

Variable	Factor Loading	Variable	Factor Loading
Information acquisition	0.822	PPB	0.653
	0.840		0.700
	0.738		0.653
Information application	0.778		0.778
	0.889		0.653
	0.792		0.739

**Table 3:** Parameter estimates.

Variance				Estimate	S.E. ^d^	*t* Value	*p* Value
IC ^e^	PPB	→	IC	0.086	0.0481	1.7863	0.0741
	PPB	→	OGB	0.6128	0.0941	6.5089	0.0000 ^c^
	IC	→	OGB	−0.3985	0.0771	−5.1684	0.0000 ^c^
IA ^f^	PPB	→	IA	0.1946	0.0534	3.6449	0.0000 ^c^
	PPB	→	OGB	0.6127	0.0959	6.3898	0.0000 ^c^
	IA	→	OGB	−0.1746	0.069	−2.5291	0.0114 ^a^

^a^ Significant at the 0.05 level (two-tailed); ^c^ significant at the 0.001 level (two-tailed); ^d^ S.E.: standard error; ^e^ IC: information acquisition; ^f^ IA: information application.

**Table 4:** Model fit.

	*X* ^2^	df	*X* ^2^_df_	GFI ^a^	AGFI ^b^	RMSEA ^c^	NFI ^d^	IFI ^e^	CFI ^f^	TLI ^g^
Figure 2-IC	126.8235	33	3.8431	0.9705	0.9509	0.0573	0.9592	0.9695	0.9694	0.9583
Figure 2-IA	155.4394	33	4.7103	0.9662	0.9437	0.0655	0.9524	0.9621	0.9620	0.9482

^a^ GFI: goodness of fit index; ^b^ AGFI: adjusted goodness of fit index; ^c^ RMSEA: root mean square error of approximation; ^d^ NFI: normed fit index; ^e^ IFI: incremental fit index. ^f^ CFI: comparative fit index; ^g^ TLI: Tucker–Lewis index.

**Table 5:** The mediation effect of EHL and the moderating effect of HL.

			Point Estimate	Product of Coefficients		Bootstrapping ^a^					
						Bias-Corrected 95% CI		Percentile 95% CI		Bias-Corrected TS ^e^	Percentile TS
				S.E.	Z	Lower	Upper	Lower	Upper		
IC	*DE* ^h^	PPB → OGT	0.6128	0.0883	6.9400	0.4415	0.7891	0.4434	0.7912	0.0005 ^d^	0.0004 ^d^
	*IE* ^I^	PPB → IC ^f^ → OGT	−0.0343	0.0233	−1.4721	−0.0861	0.0044	−0.0834	0.0074	0.0873	0.109
	*TE* ^j^	PPB → OGT	0.5785	0.0928	6.2338	0.4021	0.7675	0.4024	0.7677	0.0004 ^d^	0.0004 ^d^
IA	*DE* ^h^	PPB → OGT	0.6127	0.0906	6.7627	0.4399	0.7951	0.4415	0.7968	0.0004 ^d^	0.0004 ^d^
	*IE* ^I^	PPB → IA ^g^ → OGT	−0.034	0.0181	−1.8785	−0.0792	−0.007	−0.0743	−0.0047	0.009 ^c^	0.0166 ^b^
	*TE* ^j^	PPB → OGT	0.5787	0.0925	6.2562	0.4032	0.7677	0.4032	0.7678	0.0004 ^d^	0.0004 ^d^

^a^ Estimate from the 5000 bootstrapped samples; ^b^ significant at the 0.05 level (two-tailed); ^c^ significant at the 0.01 level (two-tailed); ^d^ significant at the 0.001 level (two-tailed); ^e^ TS = two-tailed significance; ^f^ IC = information acquisition; ^g^ IA = information application; ^h^ DE = direct effects; ^I^ IE = indirect effects; ^j^ TE = total effects.

## Data Availability

Data are contained within the article.
